# Supplementary figures and images for: Single-cell analysis reveals lasting immunological consequences of influenza infection and respiratory immunization in the pig lung
Source: PLoS Pathog. 2024 Jul 18;20(7):e1011910. doi: 10.1371/journal.ppat.1011910 (PMC11257366; doi:10.1371/journal.ppat.1011910)

Figure S1

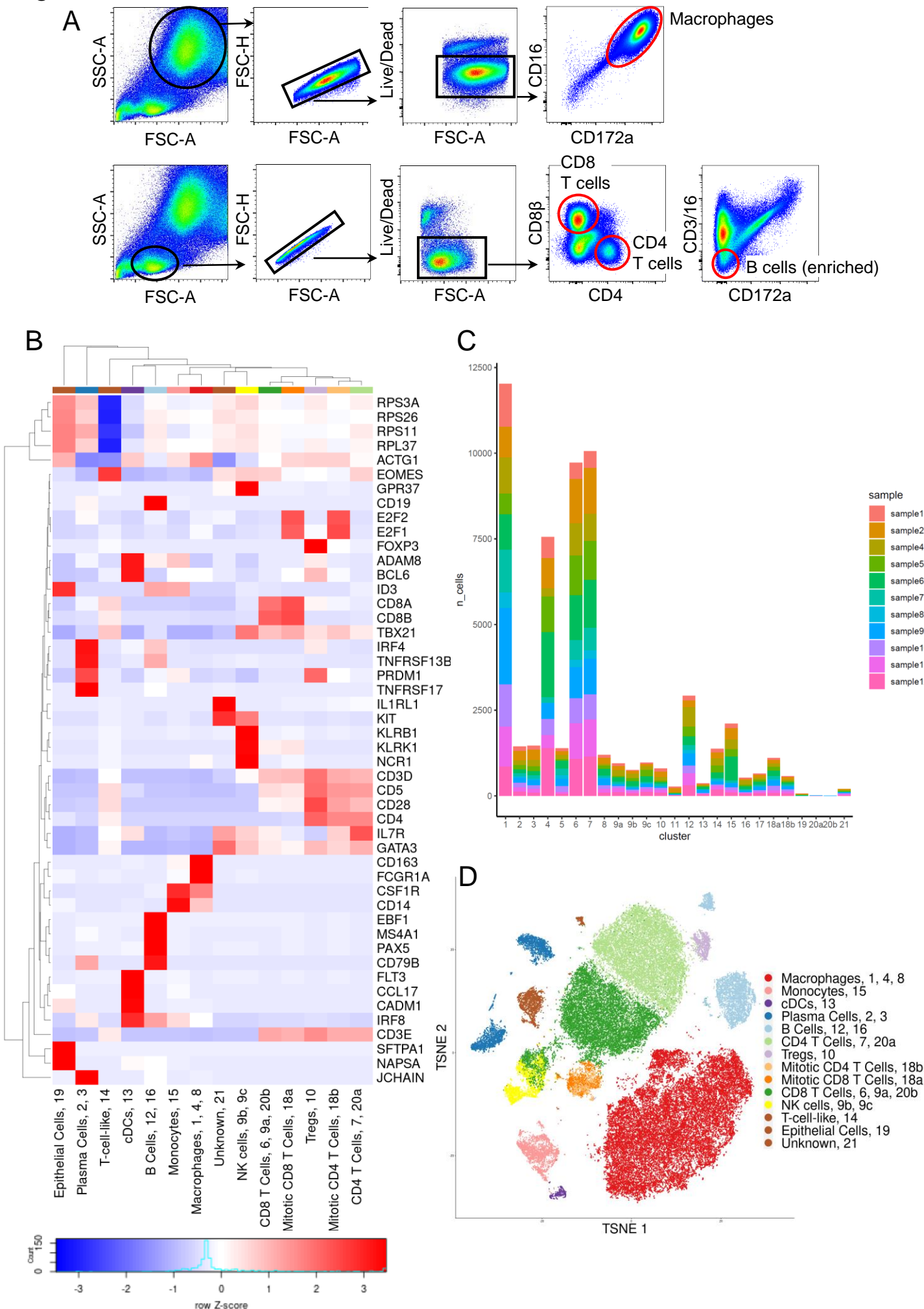

Supplement: S1 Fig — (A) Sorting strategy for balanced analysis of macrophages, CD4 T, CD8 T and B cells. Following exclusion of doublets and dead cells, macrophages were sorted based on CD172a and CD16 co-expression (top panel). CD4 T cells were sorted based on CD4 expression, and CD8 T cells based on expression of CD8β. Due to a lack of a surface pan-B cell marker for pig, B cells were enriched based on a CD3-CD16-CD172a- phenotype (bottom panel). (B) Heatmap of defining gene expression across all pig BAL scRNA-seq clusters. (C) Cluster occupancy by sample. (D) TSNE plot of pig BAL cells analysed by scRNA-seq. (PDF) [file ppat.1011910.s001.pdf]

## Figure S2

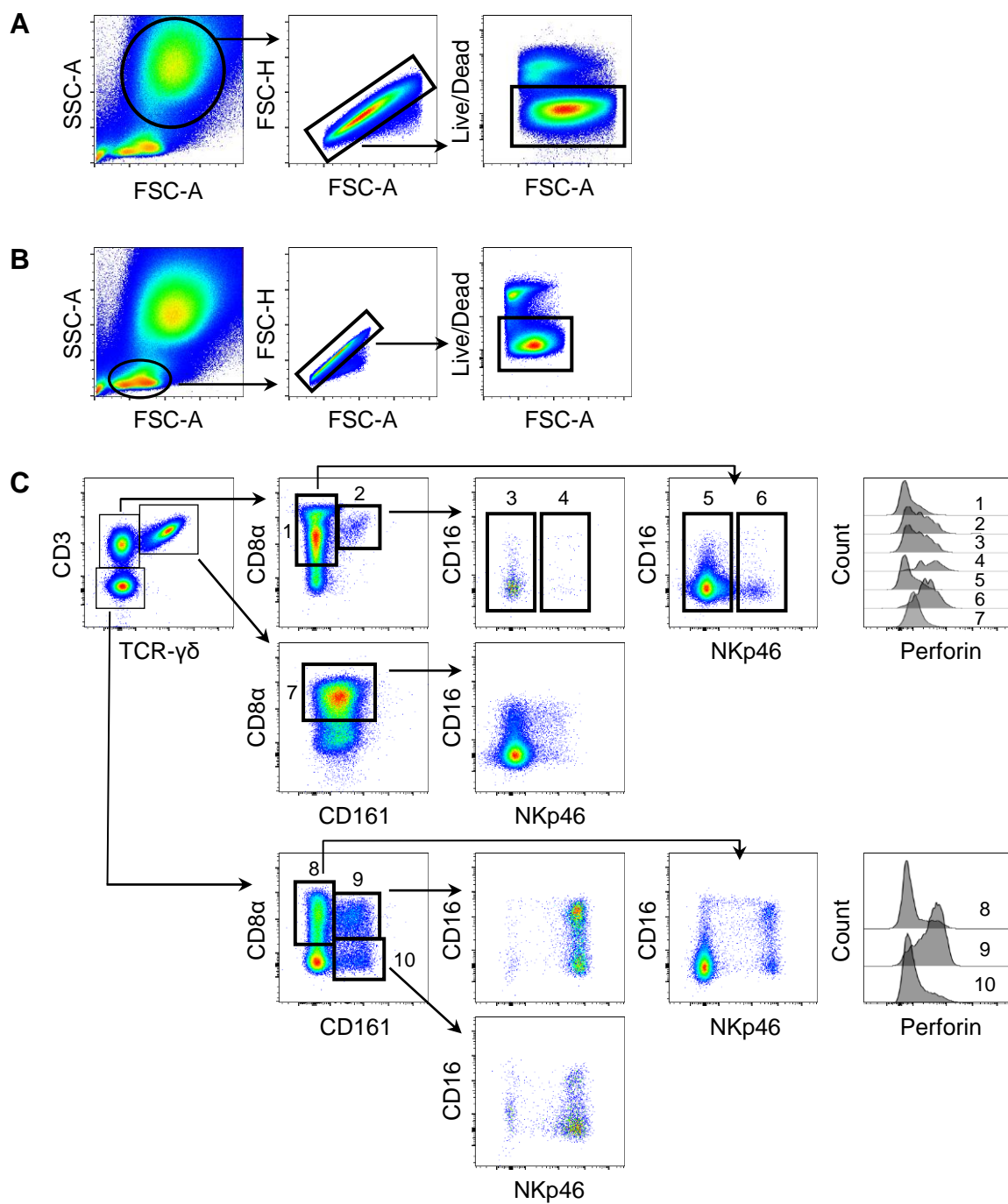

Supplement: S2 Fig — (A) For phenotyping of myeloid cells, FSC-AhighSSC-Ahigh cells were gated, followed by exclusion of doublets and dead cells (low fluorescence intensity for live/dead dye). (B) As in (A) but for phenotyping of lymphocyte subsets, initially FSC-AlowSSC-Alow cells were gated, followed by exclusion of doublets and dead cells. (C) Unconventional T cells, which had been largely excluded from scRNA-seq by the applied sorting strategy, were investigated by flow cytometry. After exclusion of dead cells and doublets as shown in (B), γδ T cells (CD3+TCR-γδ+), non-γδ T cells (CD3+TCR-γδ-) and non-T cells (CD3-TCR-γδ-) were gated and analysed for expression of CD8α, CD161, CD16, NKp46 (CD335) and perforin. (PDF) [file ppat.1011910.s002.pdf]

Figure S3

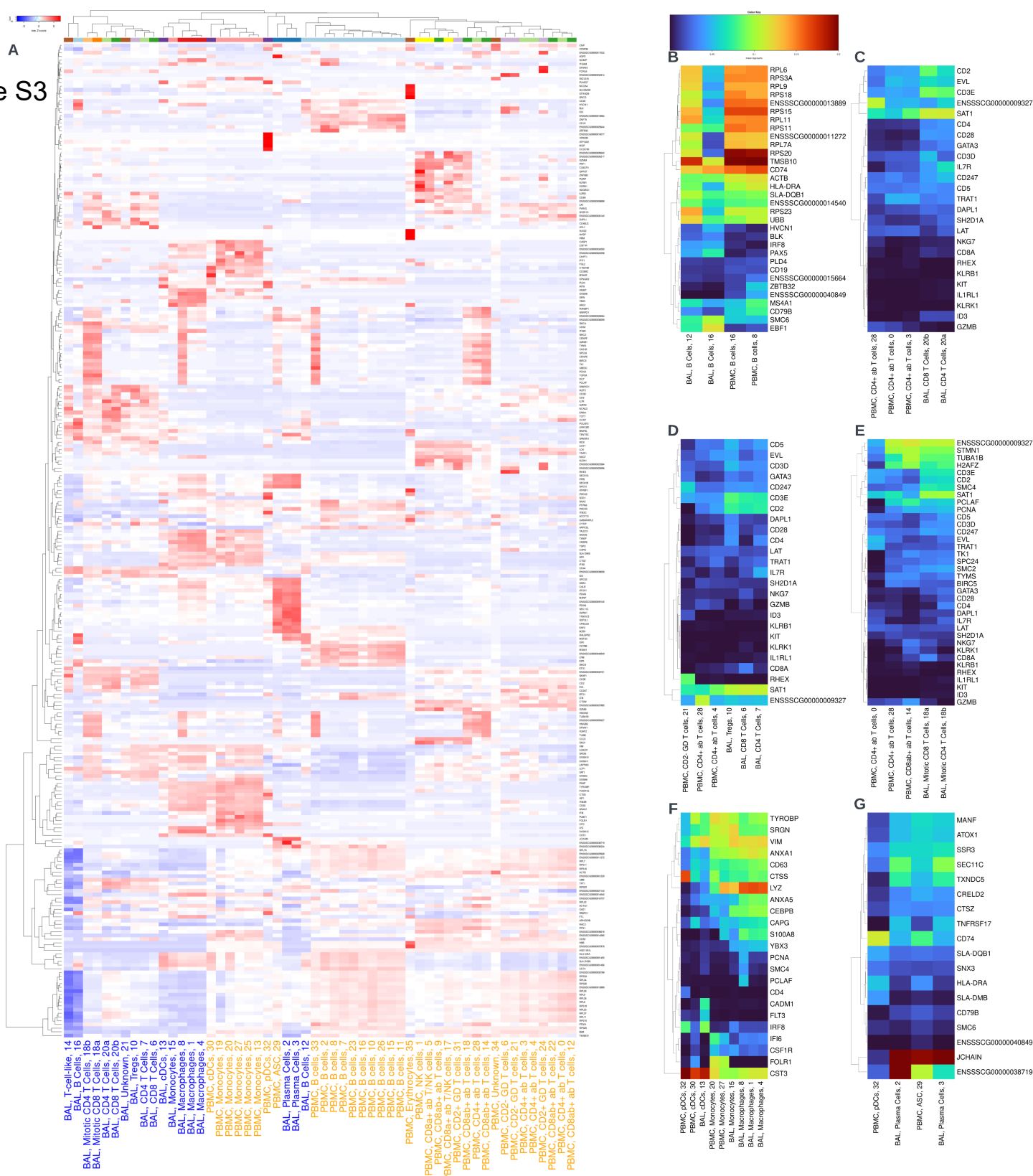

Supplement: S3 Fig — (A) Heatmap of all cell clusters and genes used in the BAL to PBMC single-cell transcriptome mapping by scMAP. (B-G) Additional heatmaps of cluster and gene subsets used in the BAL to PBMC mapping. (PDF) [file ppat.1011910.s003.pdf]

Figure S4

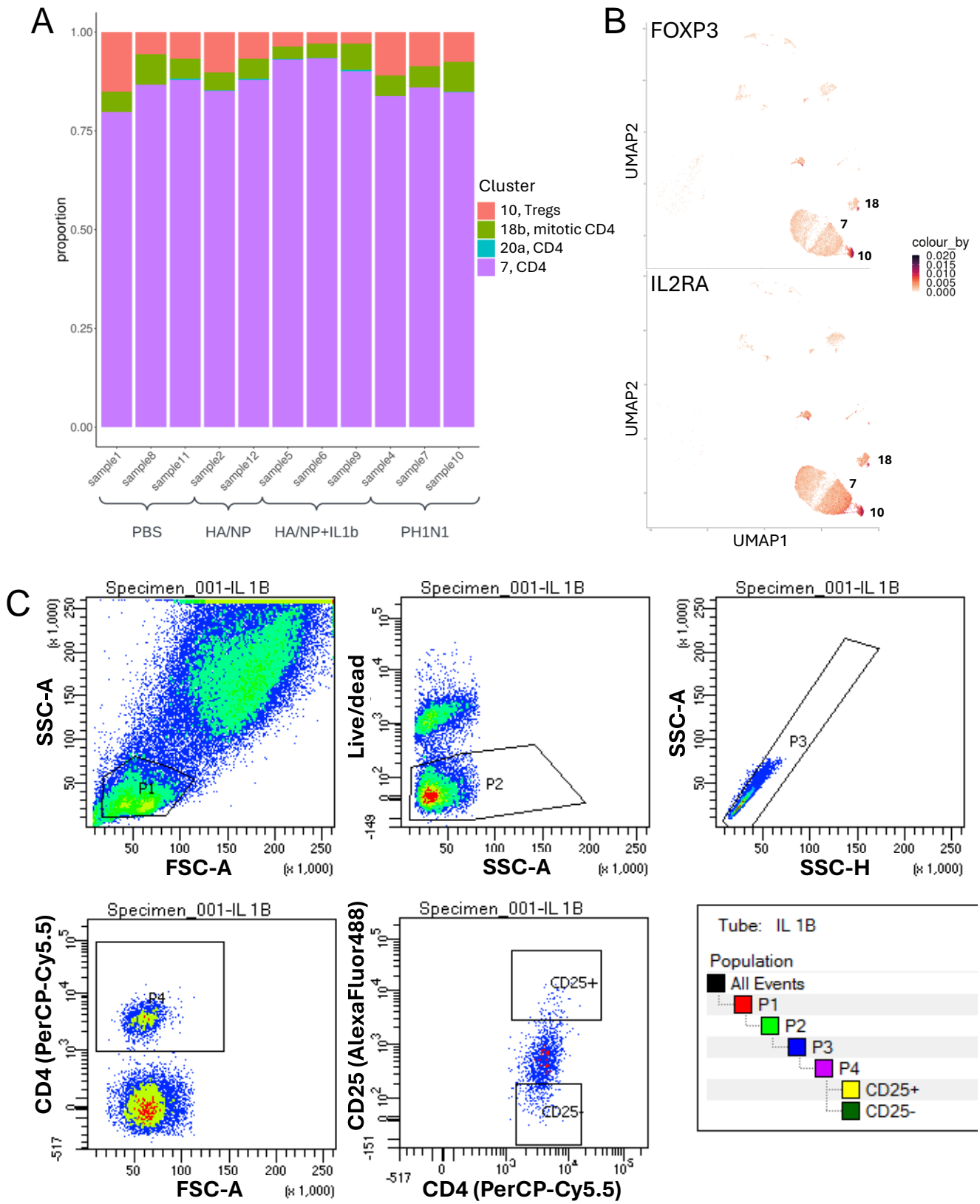

Supplement: S4 Fig — (A) Proportions of CD4 T cell clusters (7, 10, 18b, 20a) across all BAL samples in scRNA-seq data. Different treatments are indicated at the bottom of the graph. (B) UMAP plots of all cells from pig BAL scRNA-seq analysis, colored by normalised logcounts of IL2RA (CD25) and FOXP3. (C). Gating hierarchy applied for sorting of CD4+CD25- (control) and CD4+CD25high (Treg) cells from BAL of Ad-HA/NP treated pigs. (PDF) [file ppat.1011910.s004.pdf]

Figure S5

A BAL

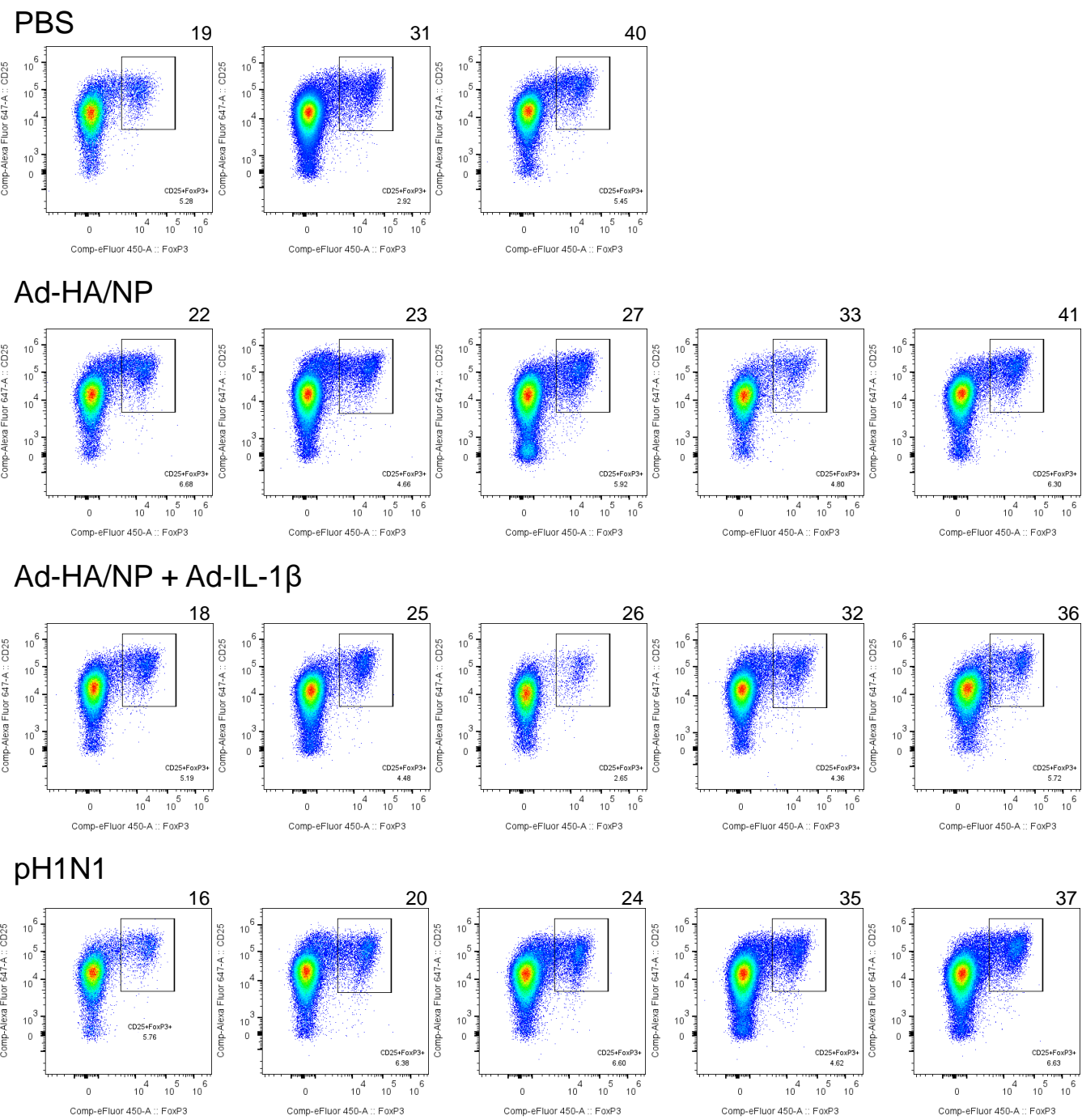

B TBLN

PBS

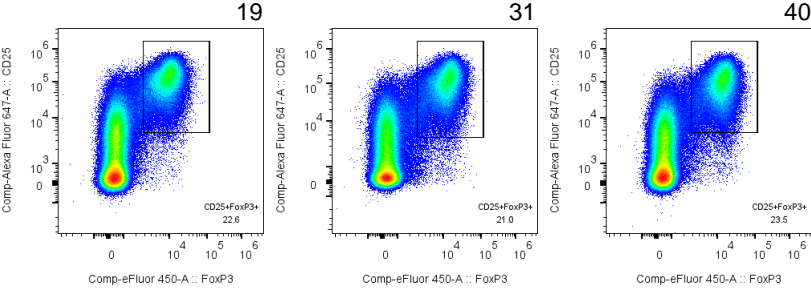

Ad-HA/NP

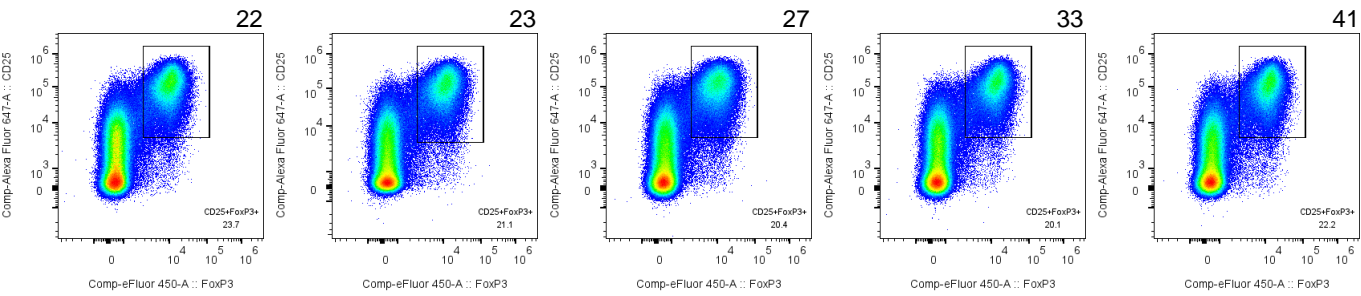

Ad-HA/NP + Ad-IL-1 $\beta$

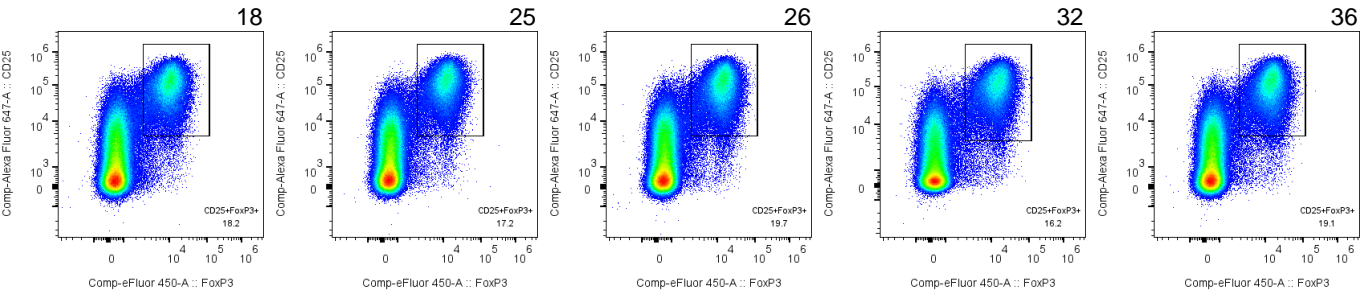

pH1N1

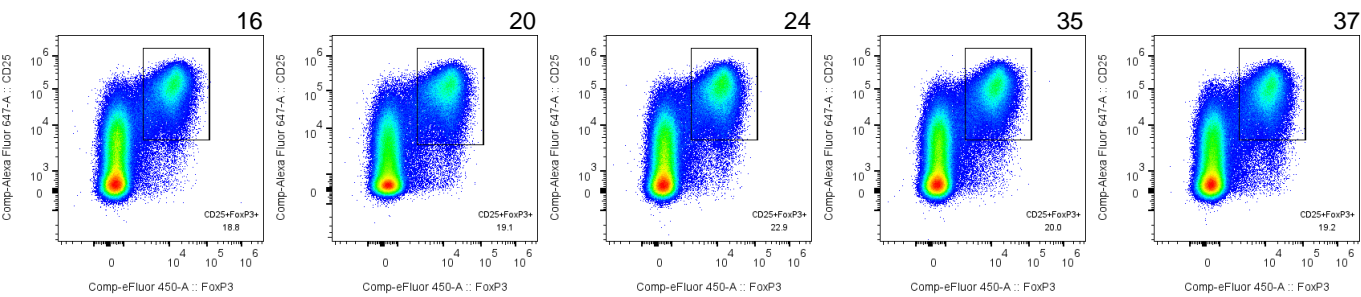

Supplement: S5 Fig — Following pre-gating on CD3+CD4+ T cells (as in Fig 2C), Treg were identified by CD25/Foxp3 co-expression. (A) BAL samples from all animals in the different treatment groups. (B) As in (A) but samples from TBLN. (A+B) The numbers in top right corners represent the animal identifiers. Percentages of CD25+Foxp3+ cells are given in the lower right corner of each pseudocolor plot. (PDF) [file ppat.1011910.s005.pdf]

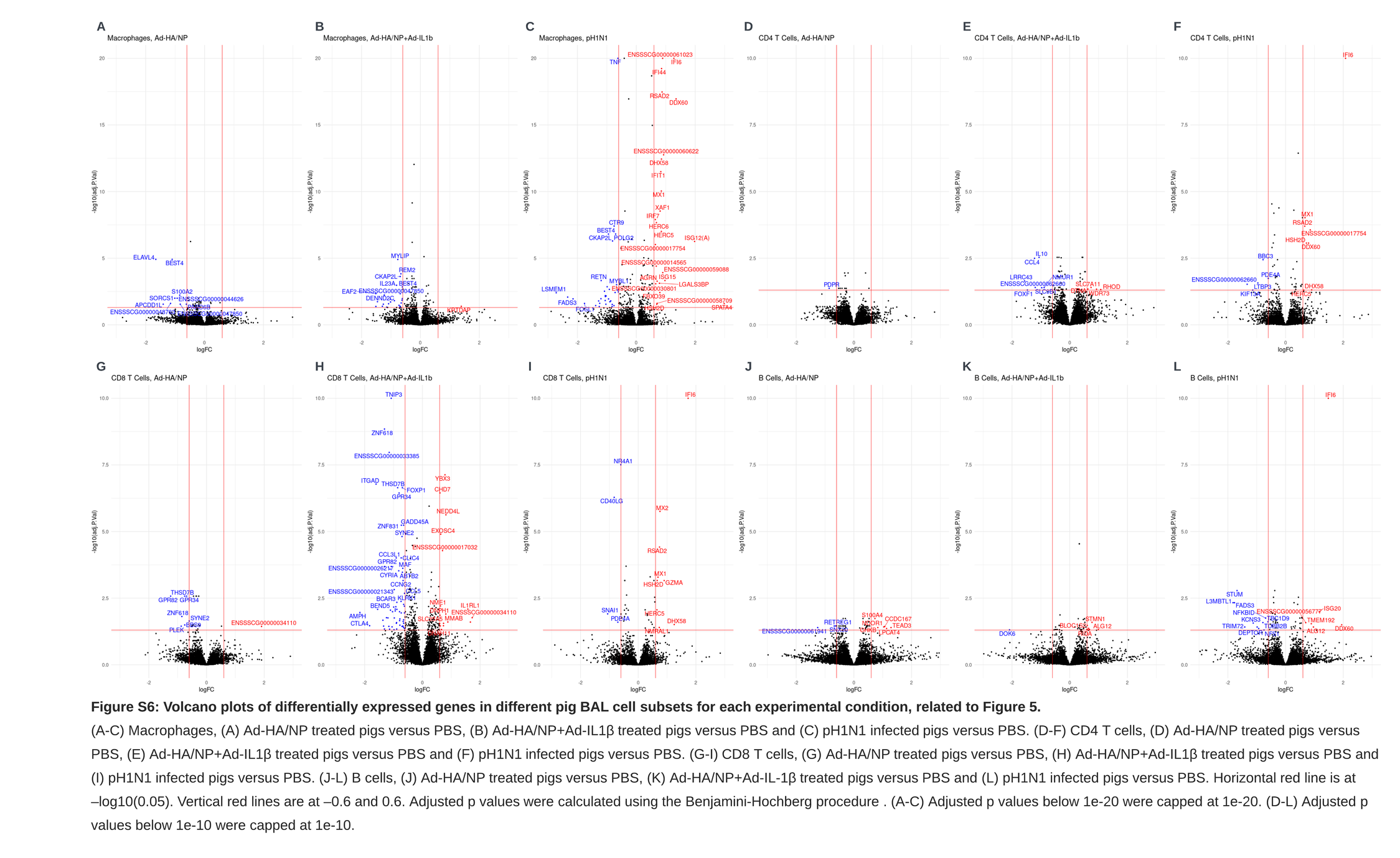

Supplement: S6 Fig — (A-C) Macrophages, (A) Ad-HA/NP treated pigs versus PBS, (B) Ad-HA/NP+Ad-IL-1β treated pigs versus PBS and (C) pH1N1 infected pigs versus PBS. (D-F) CD4 T cells, (D) Ad-HA/NP treated pigs versus PBS, (E) Ad-HA/NP+Ad-IL-1β treated pigs versus PBS and (F) pH1N1 infected pigs versus PBS. (G-I) CD8 T cells, (G) Ad-HA/NP treated pigs versus PBS, (H) Ad-HA/NP+Ad-IL-1β treated pigs versus PBS and (I) pH1N1 infected pigs versus PBS. (J-L) B cells, (J) Ad-HA/NP treated pigs versus PBS, (K) Ad-HA/NP+Ad-IL-1β treated pigs versus PBS and (L) pH1N1 infected pigs versus PBS. Horizontal red line is at–log10 (0.05). Vertical red lines are at –0.6 and 0.6. Adjusted p values were calculated using the Benjamini-Hochberg procedure. (A-C) Adjusted p values below 10−20 were capped at 10−20. (D-L) Adjusted p values below 10−10 were capped at 10−10. (TIF) [file ppat.1011910.s006.tif]

## Figure S7

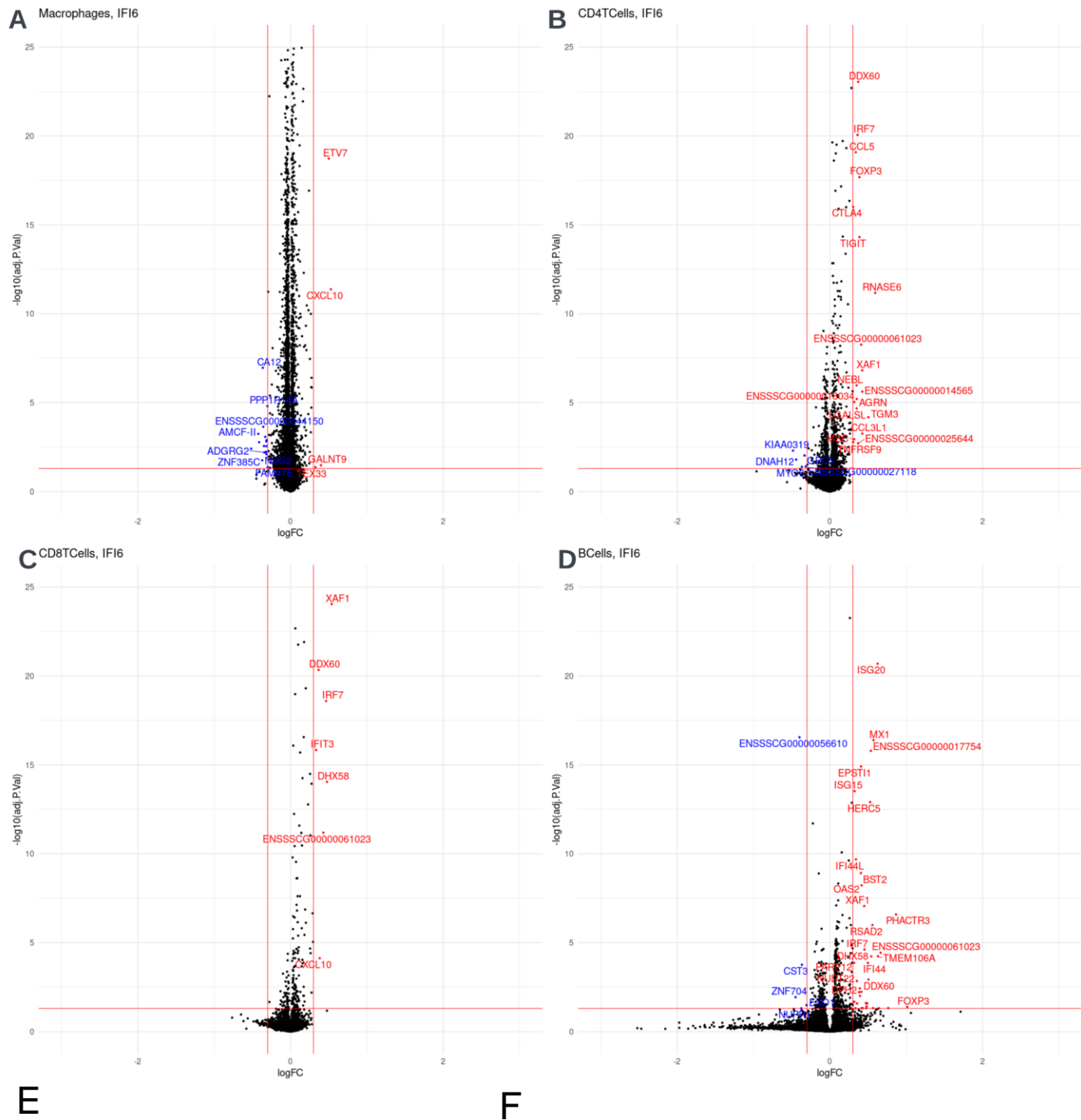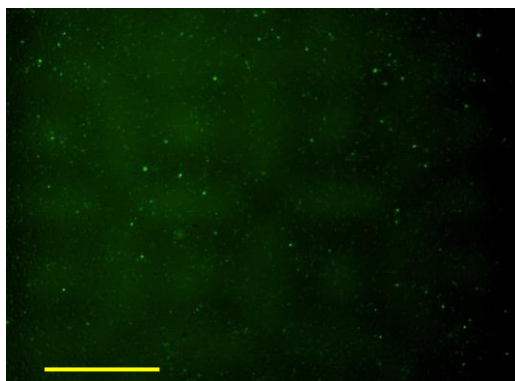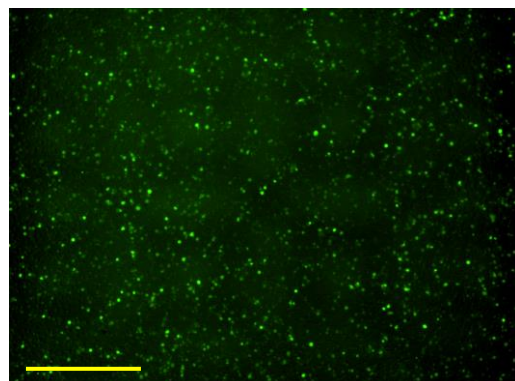

Supplement: S7 Fig — (A-D) Volcano plots of genes whose expression is associated with that of IFI6 in macrophages, CD4 T cells, CD8 T cells and B cells. Horizontal red line is at –log10(0.05). Vertical red lines are at –0.3 and 0.3. Adjusted p values were calculated using the Benjamini-Hochberg procedure. Adjusted p values below 10−100 were capped at 10−100. (A) Macrophages. (B) CD4 T cells. (C) CD8 T cells. (D) B cells. (E-F) GFP expression from infectious recombinant VLPs of vesicular stomatitis virus expressing GFP (VSVΔG-GFP) in porcine BAL cells at 7 hrs post-infection captured in live cell imager (IncuCyte-S3). Scale bars: 400nm. (E) GFP expression from VSVΔG-GFP at 7 hrs post-infection in H1N1 infected porcine BAL cells, (F) GFP expression from VSVΔG-GFP at 7 hrs post-infection in naïve (control) porcine BAL cells. (PDF) [file ppat.1011910.s007.pdf]
