# Supplementary material for: Single-cell analysis reveals lasting immunological consequences of influenza infection and respiratory immunization in the pig lung
Source: PLoS Pathog. 2024 Jul 18;20(7):e1011910. doi: 10.1371/journal.ppat.1011910 (PMC11257366; doi:10.1371/journal.ppat.1011910)
Supplement: S2 Table — Details on antibodies for each staining panel (myeloid cells, unconventional T cells, CD4 and CD8 T cells, B cells and plasma cells) are given. Second-step reagents are indicated by footnotes. (PDF) [file ppat.1011910.s009.pdf]

Supplementary Table 2: Antibodies and reagents used for FCM

Antigen	Clone	Isotype	Fluorochrome	Labelling strategy	Source primary Ab
Myeloid cells					
CD4	74-12-42	mouse IgG2b	PerCP-Cy5.5	directly conjugated	BD Biosciences
CD14	Tük4	mouse IgG2a	PE-Vio615	directly conjugated	Miltenyi Biotec
CD16	G7	mouse IgG1	AF647	directly conjugated	Bio-Rad
CD163	2A10/11	mouse IgG1	PE	directly conjugated	Bio-Rad
CD172a	74-22-15	mouse IgG1	FITC	directly conjugated	Bio-Rad
CADM1	3E1	chicken IgY	biotin	secondary antibody[1]	MBL
MHC-II (SLA-DR)	2E9/13	mouse IgG2b	BV421	secondary antibody[2]	Bio-Rad
Unconventional T cells					
CD3	BB23-8E6-8C8	mouse IgG2a	PerCP-Cy5.5	directly conjugated	BD Biosciences
CD8α	76-2-11	mouse IgG2a	BV650	biotin-streptavidin[3]	BD Biosciences
CD8β	PPT23	mouse IgG2a	PE	directly conjugated	Bio-Rad
CD16	G7	mouse IgG1	FITC	directly conjugated	Bio-Rad
CD161	1D8/10E7[4]	mouse IgG1	BV421	secondary antibody[5]	Toolbox
CD335 (NKp46)	VIV-KM1	mouse IgG1	AF647	directly conjugated	Bio-Rad
TCR-γδ	PPT16	mouse IgG2b	BUV395	secondary antibody[6]	Toolbox
T-bet	4B10	mouse IgG1	BV711	directly conjugated	BioLegend
Perforin	δG9	mouse IgG2b	PE/Dazzle 594	directly conjugated	BioLegend
CD4 and CD8 T cells					
CD3	BB23-8E6-8C8	mouse IgG2a	PerCP-Cy5.5	directly conjugated	BD Biosciences
CD4	74-12-4	mouse IgG2b	AF488	secondary antibody[7]	BD Biosciences
CD8α	76-2-11	mouse IgG2a	BV650	biotin-streptavidin[3]	BD Biosciences
CD8β	PPT23	mouse IgG2a	PE	directly conjugated	Bio-Rad
CD25	K231.3B2	mouse IgG1	AF647	directly conjugated	Bio-Rad
CD197 (CCR7)	3D12	rat IgG2a	BV711	directly conjugated	BD Biosciences
CD278 (ICOS)	C398.4A	hamster IgG	BV605	directly conjugated	BioLegend
Bcl6	K112-91	mouse IgG1	BV421	directly conjugated	BD Biosciences
Eomes	WD1928	mouse IgG1	PE-eFluor610	directly conjugated	ThermoFisher
FoxP3	FJK-16s	rat IgG2a	eFluor450	directly conjugated	ThermoFisher
Ki-67	B56	mouse IgG1	BUV737	directly conjugated	BD Biosciences
T-bet	4B10	mouse IgG1	PE-Cy7	directly conjugated	BioLegend
B cells and plasma cells					
CD21	B-ly4	mouse IgG1	BV786	directly conjugated	BD Biosciences
CD79α	HM47	mouse IgG1	PerCP-Cy5.5	directly conjugated	ThermoFisher
CD95	DX3	mouse IgG2a	SPRD	directly conjugated	Southern Biotech
Bcl6	K112-91	mouse IgG1	BV421	directly conjugated	BD Biosciences
Blimp-1	3H2-E8	mouse IgG1	AF647	directly conjugated	Santa Cruz Biotech
IRF4	3E4	rat IgG1	PE	directly conjugated	ThermoFisher
IgM	polyclonal	goat	AF680	secondary antibody[8]	Bio-Rad
Ki-67	B56	mouse IgG1	BUV737	directly conjugated	BD Biosciences
Pax5	1H9	rat IgG2a	PE-CF594	directly conjugated	BD Biosciences
T-bet	4B10	mouse IgG1	PE-Cy7	directly conjugated	BioLegend

[1]donkey anti-chicken IgY-biotin (Jackson) + Strep-BV605 (BD Biosciences)
[2]goat anti-mouse IgG2b-BV421 (Jackson)
[3]Strep-BV650 (BioLegend)
[4]This mAb was generated by the Biological Research Facility and Immunological Toolbox. Amino acid sequence of porcine CD161 used for mouse immunisations:
RQKLSIEETSMDVPESRNETTERPALLKCPTNWHPFQDKCLFFYNSYKHWNESLADCSTKESSLLLIQD

NEELRLIQNLIDSGGIIFWIGLNFSLPEENWKWINGSFLSSEILPITGVAGENNCVSISKTQMLSEPCDSENKWICQKNLKPVRNIVKK. Mouse immunisation and antibody generation was performed as described in doi: 10.3389/fimmu.2018.02246.
[5]goat anti-mouse IgG1-BV421 (Jackson)
[6]rat anti-mouse IgG2b-BUV395 (clone R12-3, BD Biosciences)
[7]goat anti-mouse IgG2b-AF488 (Jackson)
[8]donkey anti-goat AF680 (Jackson)
