# Supplementary material for: Single-cell analysis reveals lasting immunological consequences of influenza infection and respiratory immunization in the pig lung
Source: PLoS Pathog. 2024 Jul 18;20(7):e1011910. doi: 10.1371/journal.ppat.1011910 (PMC11257366; doi:10.1371/journal.ppat.1011910)
Supplement: Attachment — Submitted filename: Author response to reviewer queries.docx [file ppat.1011910.s014.docx]

**Major Issues: Key Experiments Required for Acceptance**

*Reviewer #1: The flow cytometry analysis revealed significant differences in cell population phenotype compared to scRNAseq, particularly Treg. Could this be a difference in sensitivity for the assays? That was not discussed. Also, cryopreservation has been shown to affect cell viability and binding of markers, and could be the factor resulting in differences observed. There was no information about the cryopreservation method. Please explain how the BAL was frozen and if there were any cryopreservation methods used. There should be discussion on how the freezing process could affect cell populations.*

Potential reasons for differences in Treg distribution between flow cytometry and scRNA-seq were already addressed in the previous version of the manuscript, lines 295-304 (revised manuscript: lines 295-309). To corroborate this further, we have now added two UMAP plots as subfigure B to Figure S4. These plots show FOXP3 and IL2RA (CD25) transcript expression levels across all clusters. They demonstrate that cells expressing these transcripts were present not only in cluster 10 but also in clusters 7 and 18 which contained CD4 T cells. Due to this, we argue that cells assigned as ‘Tregs’ overlap only partially for the two methods. The discussion on this (lines 295-309) has been rephrased accordingly.

Regarding the influence of cryopreservation, all cells used in this study, for flow cytometry and scRNAseq, were cryopreserved. We were able to compare frequencies of macrophages, B and T cells before and after freezing/ thawing by comparison with flow cytometry experiments described in reference 44, some of which were performed with fresh BAL cells. This showed that the frequencies of these cells were unaffected by cryopreservation, though there could be differences in more granular cell subsets. Importantly, all samples from all conditions were treated in the same manner and so any freeze/thaw effect would be shared. Information on this has been incorporated into the Discussion, lines 416 – 422. Information on the cryopreservation procedure has been added to M&M, lines 492-496.

*Data here shows that IL1beta downregulates Tregs even when compared to PBS. This could lead to a cytokine storm and more severe disease upon subsequent infection. The authors mention this quickly for their previous work data, but this should be discussed further when discussing results in Fig 4.*

We have added a sentence to clarify the role of Tregs in suppressing cytokine production and the finding that lung pathology was increased in the IL-1beta treated animals (lines 326-327).

*Reviewer #2: 1. The BAL samples were collected from 18 pigs, of which 3 from each treatment group were used for their studies. However, the authors do not clearly state in the beginning of Results, or in their M&M, whether BAL cells were pooled for their summary analyses or if the PBS treatment group was used for the basic analyses (Figs1-3). Only on l.280 do they start noting comparison of data for different treatment conditions.*

We have added in lines 150-152 (Results section) that BAL cells from each animal (3 per treatment group) were handled separately during cell partitioning and library preparation. This information has also been to M&M, lines 512-513. For clustering and cell type annotation, single-cell transcriptomes from all sequenced samples were combined (PBS and challenged, lines 154-155).

*2. L342 “IFI6 was particularly notable as being universally upregulated across leukocyte subsets.” How did the authors identify just IFI6? How many other genes were expressed across cell subsets? More data must be presented to validate the singular role of IFI6.*

IFI6 was originally identified when investigating differential expression in pH1N1 infected pigs versus PBS within macrophages, CD4 T cells, CD8 T cells and B cells (Figure 5C and 5E). IFI6 was the only differentially expressed gene to be upregulated in all four cell types. This was then further investigated within more granular cell type clusters, as illustrated in Figure 6A. Figure 5 (A-C) has been updated to make clearer the number of genes differentially expressed for each cell type and condition, with shared gene names now listed in accompanying tables D and E, the full table including shared and unshared DE genes is now available as part of Table S4. The main text has also been updated with this information (lines 329-357) as well as the legend of Figure 5 (lines 735-749).

*3. Fig,6 legend (A) IFI6 expression across clusters in each experimental condition. What actual data is this? Please clarify. How many data points for each graph? How many pigs – the 18 original? What’s the overall sex distribution?*

Figure 6A shows the expression values of IFI6 as mean log counts per million from the scRNAseq data for each cluster and treatment group. The dataset includes 11pigs, 5 males and 6 females, the same that were used for the entirety of the scRNAseq analysis. We have added information on this in the legends of Fig 6A and 4A (lines 753-754 and 727, respectively).

**Minor Issues: Editorial and Data Presentation Modifications**

*Reviewer #1: L34: single cell RNA sequencing
L314-316: “The addition of Tregs (CD4+CD25high) to the BAL nearly abolished cytokine production, while a minor effect was detected when CD4+CD25neg control cells were introduced (Figure 4C).” this sentence is a little confusing, not clear what was done since this comes prior to method. Perhaps “the incubation of Tregs with BAL cells of Ad-HA/NP+Ad-IL-1β immunized pigs nearly abolished IFNγ and TNFα production…” is a clearer way to describe results.*
Thank you, this sentence has been modified as requested (lines 319-320).

*L341: This is the first time IFI6 is mentioned in the main text. Please spell out and explain a little more of the function of IFI6 to audience who is not expert in IFN response.*

We have spelled out and explained the function of IFI6 in lines 352-354 as suggested by the reviewer. IFI6 is also discussed in more detail in the discussion section (lines 453 – 463).

*L377: How is it pulmonary immunization done? I thought this was intranasal.*

The vaccines were delivered intranasally using a mucosal atomization device (MAD) as described in Schmidt et al Front Immunol 2023 (ref 44). Using scintigraphy, we have previously shown that administration of a large volume (1 ml per nostril) using a MAD delivers part of the inoculum to the lower respiratory tract and lung, therefore the term pulmonary was used to reflect that there is deposition in the lung. However, we have changed pulmonary to respiratory immunization (line 390) to reflect more precisely the deposition of vaccines throughout the respiratory tract. We also clarified in the Materials and Methods that the administered volume for the vaccines was 2 ml per nostril and therefore reaches the lung, and we referenced the scintigraphy paper (lines 481-483).

*L382-383: Here and in many other instances, it is not clear if results mentioned are for controls, or infected, or vaccinated. That should always be specified. If referring to response in general, then state "in all treatment groups" or ‘overall”.*

We were addressing BAL cells in lines 382-383 in pigs in general. The sentence has been modified accordingly (now in line 395). Moreover, we have checked the References and the Discussion section for clarity on treatment comparisons. In line 228, we have now made it clear that the comparison of scRNA-seq data from PBMC and BAL is based on scRNA-seq data obtained from all treatment groups in our study. In lines 408-409 we have added “irrespective of treatment”. In line 428 “compared to the other treatments in our study” was added. In line 443 “of Ad-HA/NP+Ad-IL-1β immunized pigs” was added. In line 446 “following challenge infection” was added.

*L383-384: The intravenous CD3 administration should be better explained. For example: “The intravenous CD3 administration, which is the most commonly used method to define tissue residency and will only bind to circulating cells, …”*
We have clarified that intravenous CD3 administration labels only the circulating cells as suggested by the reviewer in lines 396-397.

*L435: Reduction in VSVΔG-GFP infection in BAL cells from pH1N1 infected pigs does not necessarily confirm the inhibitory role of IFI6 specifically. Many other factors could be also involved with creating the antiviral state, they may just not have been detected by you. The only way to confirm the role of IFI6 is to use knockout or suppression/inhibition studies.*

We agree with the reviewer that we have not directly assessed the specific interactions between IFI6 and VSVΔG-GFP infection. However, our data revealed that IFI6 was consistently upregulated as the predominant ISG across various cell types in pH1N1 infected pigs and therefore it is plausible that the anti-viral state observed was mainly due to IFI6 expression. Our aim was to evaluate the potential functional impact of IFI6 on VSVΔG-GFP in BAL cells from pH1N1 infected pigs without manipulating IFI6 expression artificially through overexpression or suppression. Additionally, we did not wish to passage the BAL cells or perform transfection as this would have destroyed the “natural antiviral state” present upon collection from pH1N1 infected pigs.

W*e* have now indicated in the discussion that other factors may be involved in creating the antiviral state and that the only way to show unequivocally the role of IIFI6 is by using knockout or suppression/inhibition studies (lines 458 – 459).

*Figure 3: The legend in C is too disconnected from it. The figure is very busy. It would be easier to visualize panel D if BAL/PBMC was written on the figure.*

Figure 3 has been amended such that plot C figure legend has been moved down and plot D labelled with headers.’

*Figure 4: Panel C, specify origin of cells used in the figure (HA/NP+Ad-IL-1β pigs).*

This information has been added (lines 730-731).

*Reviewer #2: 4. The authors note that all their work was performed on BAL cells that were frozen in an earlier experiment (ref. #44). The authors never provide data on freshly extracted BAL cells as a reference for their studies of the frozen samples. Without this reference the authors should more clearly note that their results are from thawed BAL samples. They should at least note that the process of storage and thawing may have resulted in loss of critical cell subsets.*

A similar query had been raised by reviewer 1. Some of the flow cytometry experiments described in reference 44 had been performed with fresh BAL cells, allowing comparison of fresh and frozen aliquots of the same sample. Comparison between fresh and frozen BAL cells did not reveal significant differences between proportions of total macrophages, T and B cells. While it is possible that particular cell subsets may be affected by liquid nitrogen storage, these effects should be shared across samples, making the data comparable if not fully comprehensive. Information on this has been incorporated into the Discussion, lines 416 – 421.

*5. Table S3 would be enhanced with an additional column noting the designated cell subset population, not just the cluster #.*

This information has been added in a revised version of Table S3.

*6. For their scRNAseq the authors have used topGO for their Gene Ontology Enrichment (l.556). They note that all results were available in Table S4. However, Table S4 is only, a hard to read listing of GO terms and associations. No % genes identified are noted. This Table should be more clearly presented, using separate columns and adding statistical values that were derived from their data analyses (l.553).*

Table S4 was originally provided as a csv file (comma separated values) due to its universal and non-proprietary format. However certain spreadsheet software (including excel) require a specific command to import csv files correctly. To suit the PLOS website’s native rendering and ease reader confusion, Table S4 has now been provided as an excel spreadsheet; csv files will still be generated from the project GitHub for anyone interested in downstream processing. Table S4 has been expanded to include separate sheets listing all differentially expressed gene names for each treatment group and cell type tested. The results from topGO include separate columns for: GO.ID, Term, Annotated (number of genes), Significant (number of genes), Expected (number of genes), classicFisher, category, dataset.% genes statistics are not provided as the values are often misleading/misinterpreted depending on how the percentages are computed, especially given the low levels of annotation in the pig (as compared to mouse or human).’

*7. The title for Figure 4 implies data interpretation. Add words “test of” in the title - Abundance and test of suppressive function of Tregs. The ordinate labels for the flow histograms in Fig.S4 should note the actual CD antigen as well as the color. That is not noted in the legend either*

The title of Fig. 4 has been modified (line 725) as requested by the reviewer. Full ordinate labels (including fluorochromes) have been added to Figure S4C. Details on the antibodies used for sorting and subsequent intracellular cytokine staining are provided in M&M, lines 636-648.

*8. For Fig 5 the exact cell clusters used to define each cell subset should be noted both in the text and figure legends. The titles for the groups analyzed, e.g. Ad-HA/NP, should be as large as the A B C denoting the graphs. Fig.5 has gene expression on a red background for macrophages; a salmon or purple color would be better. Using the red/blue color for gene names is good for gene regulation; bolding gene names would help to read the names.*

Cluster numbers have been added to both the figure legend (lines 737-738) and text (lines 332-333). Figure 5 has been reworked to improve legibility, with A-C now presenting only gene numbers with the gene names provided in accompanying tables D and E.

*9. The authors conclude that addition of Tregs (CD4+CD25high) to the BAL nearly abolished cytokine production by Tregs. Have they now reviewed their clusters [or reanalyzed BAL samples] to identify where that very high CD25+ cell subset actually cluster so that it can be measured?*

Examination of IL2RA (CD25) expression in the single cell RNAseq data reveals that cells with the highest transcript expression are strongly enriched for Treg, while also including some additional T cells from other subsets (revised Figure S4). Indeed, IL2RA was a highly ranked marker gene for delineating the Treg cluster (Table S5). We therefore believe that our use of CD25 as a surface marker for Treg enrichment in this experiment was justified and that the behaviour of the CD25high population was likely mediated by Tregs.

*10. Fig. 5D,E should be a separate figure and enlarged; they are almost impossible to read. The GO terms should be in bold lettering. The figure notes >5 genes are required to be included, but what about % of the GO term genes are differentially expressed for each comparison?*

In reworking Figure 5, panels D and E have been reallocated to F and G and doubled in plot and font size to improve legibility.

*11. Fig 5D notes GO terms not individual genes. On l.341 “… NEBULA identified 10 DE genes shared by cell-types Figure 5D).” What are those 10 genes?*

We thank the reviewer for pointing out this typo: gene names were originally displayed in 5C not 5D. In reworking Figure 5, shared gene names are now provided in 5D and E, with all genes (shared and unshared) listed in Table S4.

Additional changes:

We have made some text modifications for clarity. In addition, we have updated our differential expression analysis to account for any differences in cell subset abundance and have updated the Figures and Tables accordingly. Our main conclusions do not change.
